# Environmental Adaptation Strategies of Deep-Sea Fungi

**DOI:** 10.3390/jof12030176

**Published:** 2026-02-28

**Authors:** Shuang Leng, Chang-Hong Liu

**Affiliations:** State Key Laboratory of Pharmaceutical Biotechnology, School of Life Science, Nanjing University, Nanjing 210023, China; lengshuang@nju.edu.cn

**Keywords:** deep-sea fungi, extreme environments, adaptation mechanisms

## Abstract

Deep-sea ecosystems, characterized by extreme conditions such as high hydrostatic pressure, low temperatures, and oligotrophy, host phylogenetically and functionally diverse microbial communities. Among these, deep-sea fungi represent a critical but underexplored group whose survival strategies and adaptive mechanisms are emerging as a key research area. This review highlights recent advances in understanding how fungi adapt to deep-sea environments, focusing on strategies for managing three primary stressors: hypoxia, high pressure, and low temperature. These unique adaptations not only expand our understanding of the limits of life in extreme habitats but also offer valuable microbial resources for biotechnological innovation.

## 1. Introduction

The deep sea, defined as depths exceeding 200 m, covers over 65% of the Earth’s surface and constitutes the vast majority of the ocean, which itself occupies approximately 70% of the planet’s surface [1]. This expansive and largely unexplored realm is defined by a unique combination of extreme environmental conditions, including perpetual darkness, low temperatures, and immense pressures [2]. Geologically, the deep-sea floor is highly heterogeneous; abyssal plains make up 76% of its area, with the remainder comprising continental margins, mid-ocean ridges, seamounts, and trenches [3]. The discovery of specialized habitats such as hydrothermal vents [4], cold seeps [5], and deep hypersaline anoxic basins (DHABs) [6] has overturned the traditional view of the deep sea as a lifeless desert, revealing its capacity to support highly specialized biological communities.

In 1955, the detection of viable bacteria in abyssal pelagic sediments demonstrated that metabolically active bacterial populations could persist in sedimentary layers with geological ages exceeding one million years [7]. This finding challenged the prevailing paradigm that the deep-sea sedimentary environment was too extreme to sustain life and highlighted the remarkable tenacity of microbial life in these sediments, as well as their potential geochemical significance [3]. Subsequently, Parkes et al. (1994) proposed the existence of a highly active and globally significant prokaryotic deep biosphere beneath the seafloor sediments, estimated to account for 10% of the total carbon biomass in the global biosphere [8]. Today, it is evident that most of these microbial cells remain metabolically active, or have evolved adaptive strategies to sustain extremely low levels of metabolic activity [9,10,11,12,13]. This collective evidence establishes bacteria and archaea as crucial components of the deep-sea biosphere. In contrast, fungi, an ecologically significant group of eukaryotes, remain largely understudied in terms of their diversity in deep-sea environments.

The first accidental discovery of filamentary fungi in pelagic sediments occurred during the Mid-Pacific Expedition in 1955 [7]. Later, in 1961, Höhnk confirmed the presence of fungi formerly classified as Phycomycetes and Deuteromycetes in the deep-sea environment at a depth of 4610 m, which is considered the earliest documented record of deep-sea fungi [14]. Roth et al. (1964) later achieved the first successful isolation and identification of deep-sea fungal species, collecting *Aureobasidium pullulans* and *Dendryphiella arenaria* from water column samples at 4450 m in the subtropical Atlantic Ocean [15]. Since then, a growing body of research has documented diverse fungal species in various deep-sea environments, including subsurface sediments [16,17,18], hydrothermal vents [4,19,20], cold seeps [5,21,22], DHABs [23,24], oxygen minimum zones (OMZs) [25,26,27]. The ability of these fungi to thrive in such diverse habitats underscores their remarkable adaptability, making them ideal model systems for investigating the limits of life and the evolutionary processes of eukaryotes [28,29].

Understanding the environmental adaptation strategies of deep-sea fungi is crucial for several reasons. First, their unique adaptations provide insights into how life persists under extreme conditions, a topic of particular relevance in the context of climate change and mounting environmental stressors [28]. Additionally, the ecological roles of deep-sea fungi, including their contributions to biogeochemical cycles and their potential for biotechnological applications, highlight the importance of studying these organisms [5]. As the exploration of our oceans continues, a comprehensive understanding of deep-sea fungi will be essential for unraveling the complexities of these ecosystems and assessing their responses to global changes. This review aims to illuminate the environmental adaptation strategies employed by deep-sea fungi, emphasizing their significance in both ecological and applied contexts.

## 2. Advances in Deep-Sea Fungi Research

**Figure 1:**
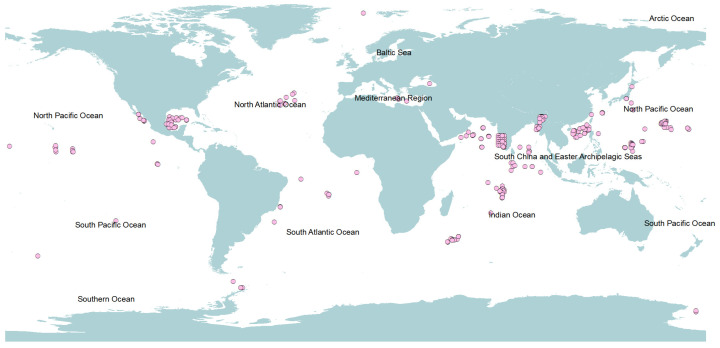
Global distribution of reported deep-sea fungi. The pink dots indicate the locations of documented fungi based on published literature, utilizing both culture-dependent and/or culture-independent methods. A Supplementary Table (Table S1) lists the studies and observations corresponding to each data point, along with relevant literature references and metadata, sorted by geographic origin in different oceans.

Research on deep-sea fungi has expanded globally, with studies spanning nearly all oceanic regions (Figure 1; Table S1). Key investigations have focused on the Pacific Ocean, particularly in the South China Sea [30,31,32], the Mariana Trench [33,34], the Yap Trench [35,36], and the Eastern Tropical Pacific [26,37]. In the Indian Ocean, significant research has been conducted in the Central Indian Basin [38,39], the Arabian Sea [27,40], and the Southwest Indian Ridge [41,42]. In the Atlantic, studies have centered on the Gulf of Mexico [43,44] and the North and South Atlantic regions [45,46]. Additional research in the Arctic Ocean [47], the Mediterranean Sea [48,49], the Red Sea [4,21], and the Southern Ocean [50] has further confirmed the global distribution and ecological importance of these organisms.

Most deep-sea fungi are found in marine sediments, while others inhabit hydrothermal vents and cold seeps, with some identified at depths of up to 2.5 km below the seafloor [22,51]. Common genera include *Aspergillus*, *Penicillium*, *Cladosporium*, *Alternaria*, and *Fusarium* [52,53,54]. Research indicates that fungi belonging to the genera *Xylaria*, *Cadophora*, and *Torulaspora* can thrive in hydrothermal vents characterized by extreme temperatures (up to 400 °C) and steep thermal gradients [51,55], whereas fungi from the genera *Fusarium*, *Suillus*, and *Cryptococcus* persist in methane-rich fluids at ambient seawater temperatures over the long term [5,56].

To investigate deep-sea fungal diversity, researchers use both traditional culture-dependent methods and modern culture-independent techniques. Although culture-dependent methods attempt to recreate deep-sea conditions for fungal isolation, they often underestimate diversity because many deep-sea fungi are difficult to culture [38,44,45,54,57,58]. Consequently, recent studies increasingly utilize total DNA extraction from sediment samples, combined with high-throughput sequencing and DNA metabarcoding [42,59,60,61,62,63,64,65,66,67]. Common genetic markers include ITS, 18S, and 28S rRNA regions, as well as protein-coding sequences such as actin and β-tubulin [36,61,68].

Current rough estimates suggest that the fungal diversity in deep-sea environments comprises at least 130 genera, based on both culture-dependent and culture-independent studies [31,36,38,42,44,45,52,53,57,59,60,62,66,67,68,69,70,71,72,73,74,75]. Prominent genera include *Aspergillus*, *Penicillium*, and *Cladosporium*, which are found across various deep-sea habitats, including hydrothermal systems, ocean trenches, seamounts, OMZs, and mid-ocean ridges [45,62,76]. Other notable genera identified in deep-sea sediments include *Fusarium*, *Exophiala*, *Candida*, and *Rhodotorula* [65,67].

Of particular interest are the seven species classified as obligate deep-sea fungi, which thrive at depths ranging from 500 to 5707 m. Five species were identified by Kohlmeyer: *Abyssomyces hydrozoicus*, found on hydrozoan substrates at depths of 631 to 641 m near the South Orkney Islands [77]; and *Allescheriella bathygena*, *Bathyascus vermisporus*, *Oceanitis scuticella*, and *Periconia abyssa*, isolated from wood substrates in Atlantic and Pacific sediments at depths ranging from 1615 to 5315 m [78]. Dupont et al. (2009) discovered *Alisea longicolla* on submerged wood at depths of 630 to 791 m off the Vanuatu Islands [79]. Most recently, *Oceanitis abyssalis* was described by Nagano et al. (2024) from a sample collected on the abyssal plain in the Northwest Pacific Ocean at a depth of 5707 m [80].

In a significant investigation by Nagano et al. (2010) examining fungal diversity in ten deep-sea sediment samples from depths between 1200 and 10,000 m, the researchers deployed three fungal-specific primer sets targeting the ITS1–5.8S–ITS2–28S rRNA regions [69]. They found that 34 out of 43 amplified ITS sequences showed minimal association with known fungal sequences in public databases. Despite this, some common surface fungi, such as *Penicillium*, *Aspergillus*, *Trichosporon*, and *Candida*, were identified. Additionally, they discovered a highly novel fungal phylotype, designated as DSF-Group1, which appears to be globally distributed in deep-sea environments but has not been reported elsewhere, except from small zooplankton (Daphnia) in European lakes [69]. Importantly, no pure cultures of DSF-Group1 have been documented to date.

## 3. Adaptive Mechanisms of Deep-Sea Fungi to Extreme Environments

Deep-sea fungi thrive in extreme environments characterized by a combination of stressors, including high hydrostatic pressure (HHP), low temperatures (2–4 °C), and frequent hypoxia or anoxia in oceanic sediments [81]. These conditions require integrated adaptive strategies that may be similar to or distinct from those employed by fungi in other environments, such as terrestrial or shallow-water habitats.

### 3.1. Hypoxia

**Figure 2:**
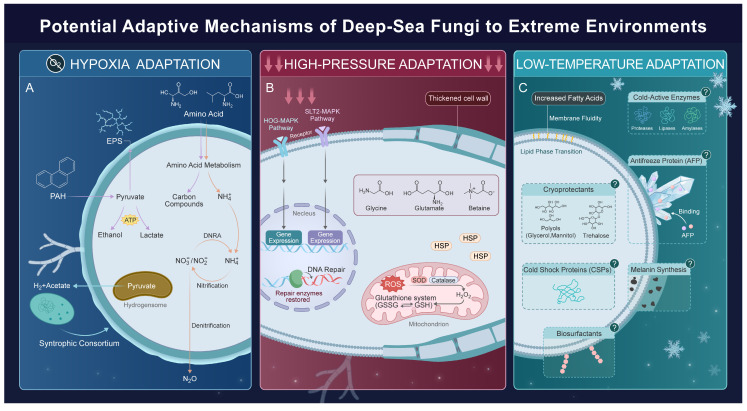
Potential adaptive mechanisms of deep-Sea fungi to hypoxia, high hydrostatic pressure, and low temperature. Panel (**A**) illustrates the adaptive responses of deep-sea fungi to hypoxic conditions through several mechanisms: formation of hydrogenosomes (brown), reprogramming carbon metabolism (pink arrow), diversification of nitrogen metabolism (orange arrow), and hydrogen-driven symbiosis (blue arrow). Panel (**B**) shows deep-sea fungi adaptation to high hydrostatic pressure via three main strategies: signal transduction and gene regulation through HOG-MAPK and SLT2-MAPK pathways that trigger adaptive gene expression and DNA repair, structural reinforcement through cell wall thickening, and maintenance of intracellular homeostasis, which involves accumulating compatible solutes, neutralizing reactive oxygen species, and deploying heat shock proteins. Panel (**C**) outlines deep-sea fungi responses to cold stress. The primary confirmed mechanism is the modification of lipid composition to maintain membrane fluidity. Other hypothetical mechanisms (dashed boxes), which are inferred from terrestrial or polar fungi, include the secretion of cold-active enzymes, synthesis of antifreeze proteins, biosurfactant and melanin, accumulation of cryoprotectants, and production of cold shock proteins. This figure was drawn using Adobe Illustrator (version 29.0.1) and Adobe Photoshop (version 20.0.0).

Deep-sea habitats, such as hadal zones (water depths exceeding 6000 m) [82] and marine sediments, are characterized by extreme hypoxic or anaerobic conditions that starkly contrast with well-oxygenated terrestrial environments. In response to these challenges, deep-sea fungi have evolved unique mechanisms to thrive despite oxygen limitations (Figure 2). One significant adaptation is the evolution of hydrogenosomes, modified mitochondria that generate ATP through fermentative metabolism rather than oxidative phosphorylation [83]. This allows fungi to flourish in permanently anoxic conditions, such as those found in the rumen of herbivorous mammals [84]. Notably, hydrogenosomes have evolved independently in various fungal lineages, including anaerobic fungi from the phylum Neocallimastigomycota found in terrestrial ruminants and certain Chytridiomycota across different environments, demonstrating convergent evolution in response to oxygen scarcity [85,86,87]. Recent discoveries also suggest that similar hydrogen-producing metabolic strategies may exist in fungi found in oxygen-depleted oceanic crust (an extreme anoxic habitat extending to ~950 m below seafloor) [88].

The metabolic byproducts of deep-sea fungi equipped with hydrogenosomes, such as hydrogen (H_2_), acetate, and formate, facilitate symbiotic interactions with prokaryotes like methanogens and sulfate reducers. These interactions create thermodynamic gradients that enhance the fungi’s fermentation efficiency [83,88]. Moreover, fungal hyphal networks act as conduits for nutrient transport in anoxic environments, enabling microorganisms to access distant resources [89,90,91]. Certain marine fungi, such as *Alternaria destruens* F10.81 and *Fusarium pseudonygamai* F5.76, can selectively translocate specific bacteria (e.g., *Spirochaeta litoralis*), creating anoxic microniches that support anaerobic bacterial survival and contribute to microbial community dynamics [92]. Additionally, fungi in the OMZ of the eastern tropical North Pacific Ocean can degrade complex polysaccharides, complementing the metabolic functions of anaerobic prokaryotes and highlighting their ecological significance in extreme anoxic habitats [93].

Deep-sea fungi, such as *Schizophyllum commune* 20R-7-F01 isolated from coal-bearing sediments ~2 km below the seafloor, exhibit a remarkable ability to degrade complex organic substrates like polycyclic aromatic hydrocarbons (PAHs), through carboxylase-mediated pathways, rather than relying on lignin-modifying enzymes like laccase (Lac), manganese peroxidase (MnP), and lignin peroxidase (LiP), which are commonly used by terrestrial fungi [94]. This specialized adaptation allows deep-sea fungi to harness energy from sources in oxygen-depleted environments, distinguishing them from their terrestrial counterparts that primarily utilize these for aerobic degradation.

Unlike most terrestrial fungi, which primarily rely on ethanol fermentation, deep-sea fungi display remarkable versatility in their anaerobic metabolic pathways. For example, *S. commune* 20R-7-F01 activates both ethanol and lactic acid fermentation pathways under anoxic conditions, creating a redundant energy generation system that enhances metabolic flexibility in oxygen-limited environments [95,96]. Additionally, deep-sea fungi can utilize nitrate as both a nitrogen source and an alternative electron acceptor during anaerobic respiration [97]. For example, *Aspergillus sydowii* DM1, isolated from the Mariana Trench, possesses complete nitrate reduction pathways, enabling it to perform denitrification in the absence of oxygen [98]. Furthermore, nearly all tested fungal species, including *Aspergillus* spp., *Cladosporium* spp., *Eutypalla* spp., *Eurotium rubrum*, *Hamigera* spp., *Penicillium* spp., *Pseudocercosporella fraxini*, and *S. commune* from deep sediments beneath the seafloor, can engage in dissimilatory nitrate/nitrite reduction to ammonium, denitrification, and nitrification under anaerobic conditions [97]. This metabolic capability optimizes nitrogen acquisition and energy production in hypoxic environments.

In the energy-scarce and low-oxygen subseafloor environments, deep-sea fungi have developed a specialized reliance on amino acids for growth, nitrogen acquisition, and as energy substrates [99]. Extensive studies on subseafloor *S. commune* 20R-7-F01 have revealed that this fungus efficiently utilizes various amino acids, particularly those with hydrocarbon chains, which are important for fruiting body formation under low-oxygen conditions [99,100]. Transcriptomic analyses indicate that amino acid metabolic pathways are upregulated under anaerobic conditions, highlighting their essential role as energy sources for survival in the deep biosphere [96]. This dependence on amino acids reduces fungi’s reliance on carbohydrates, which are often limited in deep-sea sediments [101].

Extracellular polysaccharides (EPSs), which are high-molecular-weight, sugar-based polymers secreted by microorganisms, play critical roles in stress adaptation, cellular aggregation, and interspecific interactions, particularly under extreme conditions such as high salinity, high pressure, and nutrient limitation [102]. Research has demonstrated a significant increase in EPS production under anaerobic conditions, providing the first evidence of anaerobic EPS synthesis in fungi [103]. While the exact mechanisms linking EPS to anaerobic adaptation remain unclear, it is hypothesized that EPS may enhance cellular integrity, facilitate nutrient retention, or mediate symbiotic interactions with prokaryotes [103].

### 3.2. HHP

HHP is a defining challenge of deep-sea ecosystems, with pressure increasing by 0.1 MPa for every 10 m of depth [104]. This environmental stress profoundly impacts the physiological functions of organisms, affecting cellular integrity, membrane fluidity, protein folding, DNA stability, and metabolic homeostasis [105,106,107]. While the adaptive mechanisms of prokaryotes to HHP have been extensively studied [108,109,110,111,112], the strategies employed by deep-sea fungi remain less understood. However, recent advancements in molecular and omics technologies have begun to uncover unique adaptations that enable deep-sea fungi to thrive under these extreme conditions (Figure 2).

One key adaptation involves the High-Osmolarity Glycerol (HOG)-MAPK signaling pathway, which is essential for fungal responses to various stressors [112,113,114,115]. Notably, deep-sea fungi, such as *A. sydowii* DM1 and *Purpureocillium lilacinum* FDZ8Y1, isolated from the sediments of the Mariana Trench, have developed specialized adaptations within this pathway to enhance their tolerance to HHP [98,116]. A comparative study found that *A. sydowii* DM1 exhibits exceptional piezotolerance, showing increased gene expression associated with carbohydrate metabolism and cell wall synthesis at pressures of 40 MPa, in contrast to strains of the same species from terrestrial habitats (L-5) and shallow water (SDM1). The key downstream gene hog1 showed downregulation through negative feedback, indicating three adaptive strategies: Hog1-mediated cell cycle arrest, enhanced ATP production via carbohydrate metabolism, and upregulation of pressure-responsive genes associated with DNA repair and heat shock proteins [117]. Additionally, *P. lilacinum* FDZ8Y1 integrates the HOG pathway with the SLT2 MAPK pathway, enhancing cell wall integrity monitoring and repair compared to their non-deep-sea counterparts [116].

Structural adaptations in cell walls are crucial for maintaining stability under HHP. In *A. sydowii* DM1, increased levels of glucans and mannoproteins in the cell wall contribute to internal stability [117]. Similarly, *S. commune* 20R-7-F01 upregulates chitin synthases and β-1,3-glucan synthases under high pressure, resulting in thicker cell walls [95].

To combat oxidative stress resulting from impaired mitochondrial function, deep-sea fungi utilize robust antioxidant defense mechanisms. For instance, *P. lilacinum* FDZ8Y1 upregulates antioxidant enzymes such as superoxide dismutase (SOD), catalase (CAT), and glutathione peroxidase (GPx) [116]. Similarly, *S. commune* 20R-7-F01 accumulates glutathione and activates genes encoding glutathione synthase and glutathione transferase to neutralize reactive oxygen species (ROS). It also activates specific DNA repair pathways (ATM/ATR-Chk1-CDC25) to address genetic damage induced by high pressure [95]. Additionally, *A. sydowii* DM1 synthesizes compatible solutes, including glycine, glutamate, and betaine, which are thought to help regulate osmotic balance and protect cellular proteins from denaturation under HHP [117].

Importantly, research by Deng et al. (2023) revealed that *A. sydowii* SYX6, also isolated from the sediment of the Mariana Trench, increases the production of diorcinol, an antimicrobial and cytotoxic compound, under 20 MPa [34]. Notably, its terrestrial relatives are unable to survive at such high pressures [118]. This underscores a distinctive survival strategy that deep-sea fungi employ to adapt to HHP conditions.

Certain deep-sea fungi exhibit significant metabolic responses with biogeochemical implications under HHP. For example, *S. commune* 20R-7-F01 demonstrates a remarkable increase in methane production at 35 MPa, a response attributed to pressure-induced oxidative stress that upregulates genes involved in anaerobic methane synthesis [95]. This finding highlights the potentially critical role of subseafloor fungi in global methane generation and carbon cycling. Additionally, the methane produced by these fungi may serve as a carbon source for methanotrophs within these ecosystems, thereby enriching microbial diversity and enhancing ecological interactions.

### 3.3. Low Temperature

Low temperature is a significant environmental stressor for fungi, affecting cellular integrity, membrane fluidity, protein folding, DNA stability, and metabolic homeostasis [119]. Extensive studies on terrestrial and polar fungi have shown that these organisms primarily employ several mechanisms to adapt to cold conditions, including: (1) secreting antifreeze proteins to inhibit ice crystal growth and prevent mechanical damage [120,121]; (2) accumulating osmoprotectants such as trehalose and polyols to lower freezing points and maintain osmotic pressure [122,123]; (3) synthesizing cold shock proteins that act as RNA molecular chaperones to stabilize mRNA structure and facilitate translation [124]; (4) producing psychrotolerant enzymes that function efficiently at low temperatures [125]; (5) increasing the content and unsaturation index of unsaturated lipids to maintain membrane fluidity [126]; (6) producing biosurfactants (e.g., glycolipids), which exhibit dual functionality by inhibiting ice recrystallization and serving as osmolytes to enhance cold hardiness [127,128], and (7) utilizing protective strategies such as melanin and EPS, along with ecological avoidance tactics like timing spore germination to warmer seasons [129,130].

While the cold adaptation strategies of terrestrial and polar fungi have been extensively researched, those of deep-sea fungi remain understudied. However, evidence suggests that these organisms may share similar tolerance mechanisms with their terrestrial counterparts. For example, Xu et al. (2024) reported that *Rhodotorula mucilaginosa* CTD02, isolated from a depth of 5000 m in the Yap Trench, increases the proportion of unsaturated fatty acids in its membrane from 53.83% at room temperature to 90.57% at 4 °C, which helps sustain normal biological functions under cold conditions [131]. Additionally, *Chaetomium madrasense* HM411, a symbiotic fungus found in the gut of the amphipod *Hirondellea gigas* at depths of 10,895–10,910 m in the Mariana Trench, exhibits remarkable psychrotolerant enzyme characteristics. Its endoglucanase activity at 15 °C reaches 87.5 ± 1.8 U/mL, surpassing that of its terrestrial counterpart HM412, which has an activity of 71.3 ± 3.4 U/mL, while also demonstrating a shorter stable enzyme production phase (16 days for HM411 compared to 22 days for HM412) [132]. These findings highlight the potential for deep-sea fungi to possess unique evolutionary adaptations for thriving in extreme cold environments (Figure 2). Further investigation into the specific mechanisms employed by these organisms is essential for a deeper understanding of their cold adaptation strategies.

## 4. Challenges and Future Research Directions

The deep sea, characterized by extreme conditions, hosts diverse fungal communities that have evolved complex survival strategies. Despite advances in deep-sea exploration and microbial research, our understanding of the adaptive mechanisms of deep-sea fungi remains fragmented due to various technical and methodological challenges [133]. However, emerging research, fueled by technological innovation and interdisciplinary collaboration, offers new opportunities to explore the molecular and ecological bases of fungal adaptation [134].

### 4.1. Current Challenges

A significant challenge in deep-sea fungal research is acquiring and preserving high-quality, contamination-free samples that accurately reflect the in situ physiological state of fungal communities [135]. Sampling requires specialized, costly equipment and is operationally constrained [136,137]. Maintaining environmental conditions during sample retrieval is difficult; pressure and temperature changes can induce physiological stress in fungi, leading to misinterpretation of their adaptive traits [138,139]. Additionally, contamination from terrestrial fungi poses a persistent issue [140], and variations in sediment depth complicate sample acquisition in extreme habitats like the hadal zone [141]. Although international standards for sample preservation exist, their application in fungal research remains limited [142].

Most deep-sea fungi are recalcitrant to cultivation in laboratory settings due to their adaptation to extreme, oligotrophic environments [143]. Traditional cultivation methods, which rely on nutrient-rich media and standard conditions (atmospheric pressure, room temperature, and aerobic conditions), fail to replicate the specific ecological niches these fungi inhabit. Early-diverging lineages such as Cryptomycota and Chytridiomycota may have unique adaptive mechanisms but remain unculturable with current techniques [144]. Even culturable strains present challenges in simulating the extreme multifactorial conditions, such as high pressure and low temperature, necessary to trigger their native adaptive responses [145]. Additionally, the fungi’s symbiotic or syntrophic associations with other microorganisms in their natural habitats complicate the isolation of pure cultures and hinder detailed physiological, biochemical, and genetic analyses, limiting our understanding of their specific adaptive mechanisms [146]. Notably, such syntrophic or symbiotic associations represent a key adaptive trait for microorganisms in oligotrophic extreme habitats, which further exacerbates the difficulty of pure culture isolation. For instance, fungal-bacterial syntrophy can enhance nutrient acquisition in oligotrophic habitats, with one study demonstrating this adaptive trait by showing that co-cultures of *Aeromonas* sp., *Vibrio* sp., *Coprinellus micaceus*, *Cladosporium* sp., and *Aspergillus niger* exhibited predominantly beneficial interactions under phosphorus-limited conditions, thereby facilitating microbial survival in such oligotrophic aquatic environments [147]. Similar adaptive interactions occur in other extreme environments, such as the *Candida haemulonii* complex in Thai estuaries, where interactions with free-living amoebae are associated with increased pathogenic traits [148].

Current research on deep-sea fungal adaptation primarily examines individual environmental stressors, such as high pressure or hypoxia, rather than the combined effects of multiple stressors typical in natural deep-sea habitats [149,150]. Deep-sea fungi often encounter concurrent challenges, including high pressure, low temperature, hypoxia, and anoxia, necessitating integrated adaptive strategies [151]. For instance, the growth of *Aspergillus ustus* from the Central Indian Ocean Basin is significantly influenced by hydrostatic pressure and temperature in laboratory conditions [152]. Comprehensive research is needed to understand how these fungi respond to the complex interplay of stressors they encounter in their environments.

Advances in molecular technologies such as metabarcoding, metagenomics, and transcriptomics have enhanced our ability to characterize deep-sea fungal diversity and gene expression. However, extracting high-quality nucleic acids from deep-sea samples poses challenges due to inhibitory compounds such as humic acids and heavy metals. These substances can lead to low sequencing coverage and the potential loss of genetic information associated with adaptive traits [153,154].

A significant gap in current research is the limited understanding of the adaptive mechanisms employed by obligate deep-sea fungi, such as *A. longicolla*, *A. bathygena*, *B. vermisporus*, *O. scuticella*, *P. abyssa*, *A. hydrozoicus*, and *O. abyssalis* [77,78,79,80]. The difficulty in replicating their natural habitats significantly impedes research into their physiological and biochemical pathways for adaptation. Consequently, critical insights into how these fungi cope with extreme environmental stressors remain largely unexplored, underscoring the urgent need for more focused investigations that bridge laboratory studies with the complexities of deep-sea ecosystems.

### 4.2. Future Directions

Developing high-fidelity sampling devices that maintain in situ pressure, temperature, and redox conditions during retrieval is crucial. Improving contamination control protocols will help ensure accurate community analyses [155].

To culture unculturable deep-sea fungi, researchers should create nutrient-poor media that replicate deep-sea conditions and employ co-cultivation techniques with associated microorganisms [156]. Utilizing microfluidic technologies can aid in isolating individual fungal cells for study [157,158,159].

Future studies should investigate the interactive effects of multiple stressors (e.g., pressure, temperature, hypoxia) on fungal physiology [160]. Advanced experimental systems capable of simulating natural deep-sea conditions will facilitate these investigations, particularly in extreme habitats [161].

Combining metagenomics, metatranscriptomics, metabolomics, and proteomics with functional validation methods will enhance our understanding of fungal adaptation mechanisms. Improved nucleic acid extraction techniques and gene editing technologies like CRISPR-Cas9 can facilitate the study of adaptive genes [131,162].

Investigating the relationships between deep-sea fungi, particularly obligate deep-sea fungi, and other organisms (e.g., bacteria, archaea, tube worms, shrimp, corals) is essential for understanding their adaptations [163,164]. Metagenomic analyses can identify potential symbionts, while co-cultivation experiments can reveal specific adaptive responses to symbiotic interactions [5,146].

Understanding the unique enzymes and metabolites produced by deep-sea fungi, particularly obligate deep-sea fungi that have evolved to thrive in extreme environmental conditions, can lead to innovative applications in various fields, including industry, medicine, and environmental science [165,166]. This research could drive advancements in bioprocessing, drug development, and the creation of novel biomaterials.

By addressing these challenges and prioritizing research on deep-sea fungi, particularly obligate deep-sea fungi, we can gain valuable insights into their unique adaptations and their roles within extreme ecosystems.

## 5. Conclusions

Recent advancements in deep-sea fungi research have significantly enhanced our understanding of their distribution and potential bioresources, revealing their ecological significance and biotechnological applications. These fungi exhibit unique adaptive mechanisms that allow them to thrive in extreme environments characterized by hypoxia, high hydrostatic pressure (HHP), and low temperatures. While some adaptations overlap with those of other environmental fungi, many are distinct, particularly those related to HHP and specialized metabolic pathways. Despite challenges in studying these organisms, future research should focus on integrating multi-omics technologies and innovative experimental approaches to uncover the biological processes that enable deep-sea fungi to survive and their contributions to biogeochemical cycles. Continued exploration of these fungi will deepen our understanding of life in extreme environments and unlock new biotechnological applications beneficial to fields such as medicine and environmental science.

## Data Availability

No new data were created.

## Supplementary Materials

The following supporting information can be downloaded at: https://www.mdpi.com/article/10.3390/jof12030176/s1, Table S1: Reported sampling site information of deep-sea fungi. Refs. [167,168,169,170,171,172,173,174,175,176,177,178,179,180,181,182,183,184,185,186,187,188,189,190,191,192,193,194,195,196,197,198,199,200,201,202,203,204,205] are included in the Supplementary Materials.
